# Public Knowledge of Inflammatory Bowel Diseases in Saudi Arabia: A Cross-Sectional Survey Study

**DOI:** 10.7759/cureus.40114

**Published:** 2023-06-08

**Authors:** Reem M Alqahtani, Aseel Alghanemi, Abdulrahman M Aljifri, Ibraheem M Ghulman, Saif Y Ashram, Essam A Alghamdi, Abdulrahman E Azhar, Ziad A Ibrahim, Meshal M Alsudais, Abdulaziz W Banaja

**Affiliations:** 1 Department of Family Medicine, King Abdulaziz University Hospital, Jeddah, SAU

**Keywords:** saudi arabia, ulcerative colitis, knowledge, inflammatory bowel disease, crohn’s disease

## Abstract

Background: Lack of public understanding and perception may lead to a general downplaying of inflammatory bowel disorder (IBD) symptoms as they affect a less socially acceptable area of the body, which may be a significant component in the everyday struggles of an individual with IBD.

Aim: The aim is to assess the public knowledge of Crohn's disease and ulcerative colitis in Saudi Arabia.

Method: This was an online survey study that examined public knowledge of IBD in Saudi Arabia for the duration between February and March 2023. Participants were invited to participate in this study using social media websites. The questionnaire tool comprised three sections: a sociodemographic characteristics section (seven questions), an awareness section (two questions), and a knowledge section (24 questions). A binary logistic regression analysis was utilized to identify the factors that influenced the participants’ knowledge of Crohn's disease and ulcerative colitis.

Results: A total of 630 individuals participated in this study. Around 28% of the participants reported that they had never heard of, read about, or dealt with Crohn’s disease. Around 16% of them reported that they had never heard of, read about, or dealt with ulcerative colitis. The mean overall knowledge score of the study participants was 8.3 (standard deviation: 2.4) out of 24, which is equal to 34.6% and represents a weak level of knowledge of IBD. The participants showed a weak level of knowledge for all sub-scales of knowledge related to IBD general knowledge, diet, treatments, and complications. The knowledge sub-scale level ranged between 30% and 36.7%. Females, the participants in the moderate and high-income category, those who lived in urban areas, those with a higher level of education, and those who reported having osteoarthritis were more likely to be knowledgeable about IBD compared to others (p≤0.001).

Conclusion: In Saudi Arabia, a low level of IBD awareness was identified among the general population, supporting findings from other countries. Future research should aim to identify effective educational interventions to increase public awareness of this group of diseases, which would ultimately facilitate early diagnosis and improve patient outcomes.

## Introduction

Crohn's disease and ulcerative colitis are two conditions known as inflammatory bowel disorders (IBDs) are chronic inflammatory ailments of the intestines with uncertain underlying origins. According to current thought, they are most likely the outcome of intestinal immune response dysregulation to gut microbiota components in genetically vulnerable people [1,2]. If there is no immunological response to soluble antigens in the intestinal luminal stream, this might be interpreted as a loss of oral tolerance. Although long considered to be ailments of the West, they have become a major public health concern on a worldwide scale in recent years [3-6].

For many people with IBD, their sickness is concealable or “invisible“ to others around them; thus, they do not seem to be ill. This may lead to insensitivity on the part of the general public owing to a basic misunderstanding of the condition [7,8]. This lack of public understanding and perception may lead to a general downplaying of IBD symptoms as they affect a less socially acceptable area of the body. Body image dissatisfaction (BID) has been assessed in individuals with medical conditions in which appearance-related changes and disfigurements are readily visible and interpersonally salient. It has been demonstrated that in such populations, appearance-related alterations can pose substantial obstacles to the maintenance of positive self-esteem and body image. This may further be a significant component in the everyday struggles of an individual with IBD [7,9]. Up to 84% of IBD patients suffer stigma, whereby they perceive a negative societal stereotype attached to them [10,11]. The stigma felt by individuals with IBD may decrease adherence, self-efficacy, and health-related quality of life, and increase anxiety and sadness [11,12]. Low levels of public awareness may also affect those with IBD, such as when they need immediate access to restrooms in shopping and dining facilities [13].

One earlier study has shown that an increase in public understanding, such as via media efforts, is the sole element with a positive correlation to stigma reduction [10]. A previous study found that 31% of 1,001 participants in an Austrian national poll had heard of Crohn's disease, no more than 20% had heard of ulcerative colitis, and fewer than 21% properly connected these words with intestinal illness [9]. In a poll of 1,200 people in the United States, 11% had no understanding of IBD, and the median awareness rating was 5.5/10 [10]. In contrast, public knowledge of illnesses such as diabetes is higher [9]. Participants in a study on knowledge levels regarding IBD that included patients with IBD, their friends, and family members returned results that showed levels remained very low, with a mean score of 50% [14]. Enhanced public knowledge of IBD would have the essential effect of boosting early identification and diagnosis, which may minimize primary consequences of undiagnosed illness development [9,15], in addition to the advantages of decreased stigma and increased restroom access. The noted lack of public knowledge of IBD is a basic aspect of the everyday problems experienced by adults and children with the condition, and raising awareness may lead to better outcomes and assistance with disease-related practical concerns. There are limited studies in Saudi Arabia that have examined public knowledge of IBD. Therefore, this study aimed to assess the public knowledge of Crohn's disease and ulcerative colitis in Saudi Arabia.

## Materials and methods

Study design

This study used a cross-sectional online survey using self-developed questionnaire to examine public knowledge of IBD in Saudi Arabia for the duration between February and March 2023.

Study population

Our study population comprised all the residents in Saudi Arabia who were aged 18 years and over and currently lived in the country. There were no exclusion criteria. The inclusion criteria were highlighted on the cover letter of the survey.

Participant recruitment

A convenient sampling technique was employed in this study. The participants were invited to participate in this study using social media websites (Facebook, SnapChat, and WhatsApp). The survey link was posted on Facebook, SnapChat, and WhatsApp. The study objectives were highlighted in the cover letter of the survey and the participants were informed that their participation would be considered to be written consent.

Study tool and piloting phase

The researcher developed a questionnaire tool to examine public knowledge of IBD. The questionnaire tool was developed based on an extensive literature review. The questions numbered 33. The questionnaire tool comprised three sections: a sociodemographic characteristics section (seven questions), an awareness section (two questions), and a knowledge section (24 questions). The knowledge section questions were in a yes/no format. The knowledge section was subdivided into four sub-scales. These were: general knowledge of IBD, the recommended diet, treatment, and complications. For each correct answer, a score of one was given. Therefore, the maximum attainable knowledge score would be 24. The higher the score, the better the knowledge.

The face of validity of the questionnaire was checked by expert clinicians from the faculty of medicine in King Abdul-Aziz University, Saudi Arabia and they confirmed the relevance and comprehensibility of the items in the questionnaire. They confirmed items relevance, clarity and comprehensibility, and appropriateness of response options. To check the understandability of the questionnaire tool, we conducted a pilot study on a small group of members of the general public before distributing it on a larger scale. This was performed after the questionnaire tool was checked by expert clinicians.

Ethical approval

This study was approved by the Research Ethics Committee at King Abdulaziz University (Reference Number 43-23). As participation in the study was voluntary, the research ethics committee approved the consent waiver.

Sample size

Using a confidence interval of 95%, a standard deviation (SD) of 0.5 and a margin of error of 5%, the required sample size was 385 participants.

Statistical analysis

The Statistical Package for Social Science software version 27 (IBM Corp., Armonk, NY) was used to analyze the data for this study. Descriptive statistics were used to present the findings. The normality of the knowledge score was checked using histogram and skewness and kurtosis measures. Continuous data were presented as mean and SD and categorical data were presented as frequency and percentage. Binary logistic regression analysis was used to identify factors that influenced the participants’ knowledge of Crohn's disease and ulcerative colitis. The dummy variable for the logistic regression was defined as a score of or equal to the mean score of the study sample, which was 8.3 (the cut-off point used to define good knowledge for the logistic regression analysis). A confidence interval of 95% (p < 0.05) was applied to represent the statistical significance of the results, and the level of significance was assigned as 5%.

## Results

Participants’ demographic characteristics

A total of 630 individuals participated in this study. The mean age of the study participants was 34.7 (SD: 14.7). Around 68% of them were males and 72% were employed. Almost 68% of them reported that they lived in urban areas. A similar percentage of them (64.3%) reported that they had a moderate monthly income. More than half of them (56%) reported that they had a diploma. The most common comorbidities among the study participants were osteoarthritis, hypertension, and diabetes mellitus, measuring 35.7%, 32.2%, and 27.8%, respectively. Table 1 presents the participants’ demographic characteristics.

**Table 1:** Participants’ demographic characteristics SD: standard deviation

Variable	Frequency	Percentage
Age (mean (standard deviation (sd))	34.7 (14.7) years	
Gender		
Males	428	67.9%
Employment status		
Employed	453	71.9%
Residency		
Urban	428	67.9%
Monthly income category		
Low	150	23.8%
Moderate	405	64.3%
High	75	11.9%
Education level		
High school or lower	125	19.8%
Diploma	353	56.0%
Bachelor degree	152	24.1%
History of comorbid disease		
Osteoarthritis	225	35.7%
Hypertension	203	32.2%
Diabetes mellitus	175	27.8%
Cardiovascular disease	175	27.8%
Chronic obstructive pulmonary disease	100	15.9%
Chronic kidney disease	75	11.9%
Asthma	50	7.9%
Psychological disease	50	7.9%
Rheumatoid disease	25	4.0%

Awareness of inflammatory bowel disease

Table 2 presents the participants' responses to awareness items concerning Crohn’s disease and ulcerative colitis. Around 28% of the participants reported that they had never heard of, read about, or dealt with Crohn’s disease. Around 16% of them reported that they had never heard of, read about, or dealt with ulcerative colitis.

**Table 2:** Participants' response to awareness items.

Number	Variable	Frequency	Percentage
1	“Have you ever heard or read about Crohn's disease or have you ever been dealing with this disease?”		
	“I have never heard or read about this disease”	177	28.1%
	“I have somewhere heard or seen this term”	126	20.0%
	“I have already gained some information about this disease, read up on it”	75	11.9%
	“I have already dealt with it myself or within family and friends”	77	12.2%
	“I don't know”	175	27.8%
2	“Have you ever heard or read about ulcerative colitis or have you ever been dealing with this disease?”		
	“I have never heard or read about this disease”	101	16.0%
	“I have somewhere heard or seen this term”	127	20.2%
	“I have already gained some information about this disease, read up on it”	75	11.9%
	“I have already dealt with it myself or within family and friends”	75	11.9%
	“I don't know”	252	40.0%

Participants’ knowledge of inflammatory bowel syndrome

The mean overall knowledge score of the study participants was 8.3 (SD: 2.4) out of 24, which is equal to 34.6% (ranged between 17% and 54%), and represents a weak level of knowledge of IBD. The participants showed a weak level of knowledge for all the sub-scales of knowledge related to IBD general knowledge, recommended diet, treatments, and complications. The knowledge sub-scale level ranged between 30% and 36.7%. For further details on the knowledge score for each sub-scale refer to Table 3.

**Table 3:** Knowledge score stratified by sub-scale.

Subscale category	Mean score (standard deviation)	Percentage from total score for the subscale
General IBD knowledge (11 questions)	4.0 (1.3)	36.4%
Diet (2 questions)	0.6 (0.6)	30.0%
Treatments (5 questions)	1.6 (1.1)	32.0%
IBD complications (6 questions)	2.2 (1.0)	36.7%

Females, participants in the moderate and high-income category, those who lived in urban areas, those with a higher level of education, and those who reported having osteoarthritis were more likely to be knowledgeable about IBD compared to others (p≤0.001) (Table 4).

**Table 4:** Binary logistic regression analysis to identify predictors of knowledge.

Variable	Odds ratio (95% confidence interval)	P-value
Age category		
34.7 years and lower (Reference category)	1.00	
Above 34.7 years	0.57 (0.42-0.79)	p≤0.001
Gender		
Males (Reference category)	1.00	
Females	7.4 (5.0-10.8)	p≤0.001
Employment status		
Unemployed (Reference category)	1.00	
Employed	0.06 (0.04-0.10)	p≤0.001
Residency		
Rural (Reference category)	1.00	
Urban	7.4 (5.0-10.8)	p≤0.001
Monthly income category		
Low (Reference category)	1.00	
Moderate	1.99 (1.42-2.79)	p≤0.001
High	2.89 (1.74-4.81)	p≤0.001
Education level		
High school or lower (Reference category)	1.00	
Diploma	1.69 (1.22-2.33)	0.001
Bachelor degree	3.53 (2.40-5.20)	p≤0.001
History of comorbid disease		
Hypertension	1.37 (0.98-1.92)	0.065
Diabetes mellitus	0.13 (0.09-0.21)	p≤0.001
Chronic kidney disease	0.60 (0.36-1.00)	0.050
Cardiovascular disease	0.94 (0.66-1.34)	0.728
Asthma	-	-
Chronic obstructive pulmonary disease	-	-
Osteoarthritis	2.08 (1.49-2.90)	p≤0.001
Rheumatoid disease	-	-
Psychological disease	-	-

## Discussion

The key findings of this study are as follows: 1) a considerable proportion of the Saudi population reported that they had never heard of, read about, or dealt with Crohn’s disease or ulcerative colitis; 2) the general public in Saudi Arabia demonstrated a weak level of knowledge of IBD. The participants showed a weak level of knowledge for all the sub-scales of knowledge related to IBD general knowledge, recommended diet, treatments, and complications, and 3) females, participants in the moderate and high-income category, those who lived in urban areas, those with a higher level of education, and those who reported having osteoarthritis were more likely to be knowledgeable about IBD compared to others. Few studies have examined public knowledge of IBD. These have focused on specific populations, including patients with IBD and pediatric populations [16-19].

Although the incidence of IBD has been stable (or perhaps dropping) in many Western countries, prevalence is increasing owing to the disease's early onset and low mortality [6]. IBD is becoming prevalent in populations in newly Westernized countries at a pace equivalent to the substantial increases witnessed in North America, Europe, and Oceania during the previous century. IBD places a substantial financial and resource strain on healthcare systems [6]. The reason for IBDs is unknown. They are thought to be the outcome of dysregulation of the intestinal immune response to gut microbiota components in genetically vulnerable people [2,20]. This might be interpreted as a loss of oral tolerance, or the absence of an immunological response to soluble antigens in the intestinal luminal stream [21]. Dietary shifts and environmental pathogen exposure are additional contributors to the increase in IBD prevalence [21].

In our study, we found that 28% of the participants reported that they had never heard of, read about, or dealt with Crohn’s disease. Around 16% of them reported that they had never heard of, read about, or dealt with ulcerative colitis. This is lower than the findings of a previous study in Austria, which reported that 68% to 79% of the participants had never heard of or read about IBDs [9]. The mean overall knowledge score for our study participants was 8.3 (SD: 2.4) out of 24, which is equal to 34.6% and represents a weak level of knowledge of IBD. The participants showed a weak level of knowledge for all the sub-scales of knowledge related to IBD general knowledge, recommended diet, treatments, and complications. A previous study in New Zealand reported a higher level of knowledge of IBD compared to our study (58%) [19]. Similarly, this study reported a deficiency in all knowledge sub-scales (treatment, growth, diet, and risk factors) [19]. Another study in Austria also reported a weak level of knowledge of IBD among the Austrian population [9]. A previous systematic review reported that there is a lack of knowledge about IBDs among both members of the general public and patients [22]. An inadequate level of public knowledge of IBD may have multiple negative consequences on patients' outcomes. It might lead to disease progression as a consequence of delayed medical consultation [9,19]. Increasing public awareness of IBD could lead to an increase in self-referrals to general practitioners, resulting in early diagnosis and the prompt initiation of effective treatment. The beliefs and knowledge of patients regarding their disease may affect disease management, quality of life, and psychological health related to it [22]. Low treatment adherence is associated with therapy-related misinformation [22]. To facilitate earlier diagnosis, mass media, and education campaigns may increase public awareness [22].

In our study, we found that females, participants in the moderate and high-income category, those who live in urban areas, those with a higher level of education, and those who reported having osteoarthritis were more likely to be knowledgeable about IBD compared to others (p≤0.001). This was different from the findings of a previous study in New Zealand, which reported that there was no statistically significant difference in the level of knowledge among participants from different demographic groups [19]. Previous studies have identified that a higher level of education is associated with a higher level of knowledge of IBD [16-18].

The use of an online survey to recruit the study participants is not free from criticism as a considerable proportion of the targeted population could be individuals who do not have access to social media websites. However, according to the last available statistics in 2023, the number of social media users in Saudi Arabia is around 79.3% of the total population.

## Conclusions

Confirming the findings from other countries around the world, a low level of knowledge of IBD was identified among the general public in Saudi Arabia. Future studies should aim at identifying effective educational interventions to enhance public knowledge of this class of diseases, which may ultimately facilitate early diagnoses and patients’ outcomes positively.

## Appendices

Questionnaire tool:

1-     Sociodemographic Characteristics- البيانات الاجتماعية والديموغرافية

Age

العمر

Gender

الجنس

Male

ذكر

Female

أنثى

Age category

الفئة العمرية

18-20 Years

18-20 سنة

21-39 Years

21-39 سنة

40-59 Years

40-59 سنة

60 or More

60 سنة فأكثر

Employment

الوظيفة

Yes

موظف

No

غير موظف

Residency

مكان الإقامة

Urban

مدني

Rural

ريفي

Monthly income

الدخل الشهري

Weak

قليل

Good

متوسط

High

مرتفع

Educational level

المستوى التعليمي

Illiterate

أمي

High school or less

الثانوية العامة فأقل

University level

الجامعة

Have you been diagnosed with IBD previously?

هل تم تشخيصك سابقًا بمرض التهاب الأمعاء (مرض كرون أو التهاب القولون التقرحي)؟

Yes

نعم

No

لا

Do you have any relative been diagnosed with IBD previously?

هل لديك أي قريب تم تشخيصه سابقًا بمرض التهاب الأمعاء (مرض كرون أو التهاب القولون التقرحي)؟

Yes

نعم

No

لا

Comorbidity

الأمراض المزمنة

Hypertension

ارتفاع ضغط الدم

Diabetes Mellitus

مرض السكري

Chronic Kidney disease

مرض الفشل الكلوي المزمن

Cardiovascular disease

أمراض القلب والأوعية الدموية

Asthma

مرض الربو

COPD

مرض الانسداد الرئوي المزمن

Osteoarthritis

خشونة المفاصل

Rheumatoid disease

مرض الروماتيزم

Psychological disease

مرض نفسي

Others

غيرها

2-     Awareness about IBD  الوعي حول مرض التهاب الأمعاء (مرض كرون أو التهاب القولون التقرحي)

Have you ever heard or read about Crohn's disease or have you ever been dealing with this disease?

هل سبق لك أن سمعت أو قرأت عن مرض كرون أو هل سبق لك التعامل مع هذا المرض؟

1)     I have never heard or read about this disease.

1) لم أسمع أو أقرأ عن هذا المرض من قبل.

2)     I have somewhere heard or seen this term.

2) لقد سمعت أو رأيت هذا المصطلح في مكان ما.

3)     I have already gained some information about this disease, read up on it.

3) لقد حصلت بالفعل على بعض المعلومات حول هذا المرض ، قرأت عنها.

4)     I have already dealt with it myself or within family and friends.

4) لقد تعاملت بالفعل مع مرض كرون بنفسي أو خلال تعاملي مع العائلة والأصدقاء.

5)     Don't know

5) لا أعلم

Have you ever heard or read about ulcerative colitis or have you ever been dealing with this disease?

هل سمعت أو قرأت عن التهاب القولون التقرحي أو هل سبق لك التعامل مع هذا المرض؟

6)     I have never heard or read about this disease.

1) لم أسمع أو أقرأ عن هذا المرض من قبل.

7)     I have somewhere heard or seen this term.

2) لقد سمعت أو رأيت هذا المصطلح في مكان ما.

8)     I have already gained some information about this disease, read up on it.

3) لقد حصلت بالفعل على بعض المعلومات حول هذا المرض ، قرأت عنها.

9)     I have already dealt with it myself or within family and friends.

4) لقد تعاملت بالفعل مع مرض التهاب القولون التقرحي بنفسي أو خلال تعاملي مع العائلة والأصدقاء.

10) Don't know

5) لا أعلم

3-     Knowledge about IBD المعرفة حول مرض التهاب الأمعاء (مرض كرون أو التهاب القولون التقرحي)

Item

Yes

نعم

No

لا

Don’t know

لا أعلم

General IBD knowledge (11 questions)

معلومات عامة

Proctitis is a form of colitis that affects the rectum or back passage only.

التهاب المستقيم هو شكل من أشكال التهاب القولون الذي يصيب المستقيم أو فتحة الشرج فقط.

Being symptom-free for three years does not mean IBD is cured.

كونك خاليًا من الأعراض لمدة ثلاث سنوات لا يعني أن مرض التهاب الأمعاء قد تم علاجه.

IBD runs in families.

داء التهاب الأمعاء متوارث في العائلات.

The terminal ileum is a section of the bowel just before the anus.

الدقاق هو جزء من الأمعاء قبل فتحة الشرج مباشرة.

During a flare-up of IBD, the platelet count in the blood rises.

أثناء تفاقم مرض التهاب الأمعاء، يرتفع عدد الصفائح الدموية في الدم.

Ulcerative colitis is common in Europeans and North Americans.

التهاب القولون التقرحي شائع عند الأوروبيين والأمريكيين الشماليين.

The length of the small bowel is approximately 6 m.

يبلغ طول الأمعاء الدقيقة حوالي 6 أمتار.

The function of the large bowel is to absorb water.

وظيفة الأمعاء الغليظة هي امتصاص الماء.

Another name for an ileorectal anastomosis operation with formation of a reservoir is pouch.

الاسم الآخر لعملية المفاغرة اللفائفية المستقيمية هو الجيبة الشرجية

There are millions of tiny "hairs" in the small bowel to increase the absorptive surface, which are called villi.

. هناك الملايين من "الشعيرات" الدقيقة في الأمعاء الدقيقة لزيادة سطح الامتصاص، والتي تسمى الزغابات.

Headache is not a common symptom of IBD.

الصداع ليس من الأعراض الشائعة لمرض التهاب الأمعاء.

Diet (2 questions)

الغذاء

Patients are allowed to eat dairy products.

يسمح للمرضى بتناول منتجات الألبان.

Elemental feeds are very easy to digest.

الأغذية الأولية سهلة الهضم

Treatments (5 questions)

العلاجات

Steroids can be given in the form of an enema into the back passage.

يمكن إعطاء الكورتيزون في شكل حقنة شرجية.

Immunosuppressive drugs are given to IBD patients to reduce inflammation in the bowel.

يتم إعطاء الأدوية المثبطة للمناعة لمرضى أمراض التهاب الأمعاء لتقليل الالتهاب.

Sulfasalazine is used to reduce the frequency of flare-ups.

يستخدم سلفاسالازين لتقليل تكرار نوبات تفاقم المرض

Azathioprine is an immunosuppresive drug.

الآزوثيوبرين دواء مثبط للمناعة.

Male patients who take sulfasalazine have reduced fertility levels that are reversible.

المرضى الذكور الذين يتناولون سلفاسالازين قد يعانوا من قلة مستويات الخصوبة التي غالبًا ما تكون مؤقتة.

IBD complications (6 questions)

مضاعفات المرض

Inflammation can occur in other parts of the body as well as the bowel.

يمكن أن يحدث الالتهاب في أجزاء أخرى من الجسم فضلًا عن الأمعاء.

A fistula is an abnormal track between two pieces of bowel or between the bowel and skin.

الناسور هو مسار غير طبيعي بين قطعتين من الأمعاء أو بين الأمعاء والجلد.

A woman with Crohn's disease may find it more difficult to become pregnant.

قد تجد المرأة المصابة بداء كرون صعوبة أكبر في الحمل.

If terminal ileum is removed during surgery, the patient will have impaired absorption of vitamin B12.

إذا تمت إزالة الدقاق الطرفي أثناء الجراحة، فسوف يعاني المريض من ضعف امتصاص فيتامين ب 12.

Patients with IBD which has lasted for 8-10 years need to be screened for cancer of the colon.

المرضى الذين يعانون من مرض التهاب الأمعاء الذي استمر لمدة 8-10 سنوات يحتاجون إلى فحص سرطان القولون.

A child who has IBD probably will not be as tall as his or her friends.

من المحتمل ألا يكون الطفل المصاب بمرض التهاب الأمعاء طويل القامة مثل أصدقائه.
